# Biocompatibility and Antibacterial Action of *Salvadora persica* Extract as Intracanal Medication (In Vitro and Ex Vivo Experiment)

**DOI:** 10.3390/ma15041373

**Published:** 2022-02-12

**Authors:** Samah Samir Abdeltawab, Tariq S. Abu Haimed, Hammam Ahmed Bahammam, Wafaa Talat Arab, Ensanya A. Abou Neel, Laila Ahmed Bahammam

**Affiliations:** 1 Endodontic Department, King Abdulaziz University, Jeddah 21589, Saudi Arabia; dr.s.s.abdultawab@gmail.com; 2 Operative Dentistry, King Abdulaziz University, Jeddah 21589, Saudi Arabia; tabuhaimed@kau.edu.sa; 3 Department of Pediatric Dentistry, King Abdulaziz University, Jeddah 21589, Saudi Arabia; habahammam@kau.edu.sa; 4 King Fahd Medical Research Center, King Abdulaziz University, Jeddah 21589, Saudi Arabia; wtarab@kau.edu.sa; 5 Preventive and Restorative Dentistry Department, College of Dental Medicine, University of Sharjah, Sharjah 27272, United Arab Emirates; 6 Biomaterials Department, Faculty of Dentistry, Tanta University, Tanta 31527, Egypt; 7 UCL Eastman Dental Institute, Biomaterials & Tissue Engineering, Royal Free Hospital, Rowland Hill Street, London NW32QG, UK; 8 Department of Endodontics, Faculty of Dentistry, King Abdulaziz University, Jeddah 21589, Saudi Arabia

**Keywords:** *S. persica*, miswak, Ca(OH)_2_, *E. faecalis*, intracanal medication, arak roots

## Abstract

This study aimed to test the biocompatibility and antibacterial properties of *Salvadora* *persica* (*S. persica*) extract, a natural product, as an intracanal medication in comparison with calcium hydroxide (Metapaste, META BIOMED, Cheongju, Korea). The pH values of both materials were tested. The biocompatibility of *S. persica* extract and Metapaste was determined using light microscopy and MTT assays. The antibacterial action was tested using the zone of bacterial inhibition on four common bacterial species. In addition, intracanal medication was administered using 68 extracted single-rooted teeth contaminated with *Enterococcus faecalis (E. faecalis)*, and the percentage reduction in colony count (% RCC) at 1, 3, and 7 days was measured. The extension of activity for both materials was assessed using histological sections and scanning electron microscopy. *S. persica* was found to be acidic in nature. Both materials showed significantly lower cell viability than the positive control cells on days 1 and 3 but not on day 7. *S. persica* showed better antibacterial effects against *E. faecalis* and *S. mutans*. *S. persica* extract showed 97.6%, 98.9%, and 99.3% RCC values at 1, 3, and 7 days, respectively, which are comparable to those of Metapaste. *S. persica* herbal extract is a promising material that can be utilized as an intracanal medication, but its use requires further research.

## 1. Introduction

During root canal treatment, the conventional mechanistic approach of cleaning and shaping cannot achieve the desired removal of bacteria penetrating the dentinal tubules [1]. Furthermore, 9.6–47.6% of the root canal is left untouched after mechanical instrumentation [2,3], which is covered with pulp tissue remnants, bacteria, and dentin chips [4], acting as a nidus for reinfection [5]. The use of additional chemical reagents in the form of inter-procedure irrigation solutions or intracanal medications is advocated to enhance the cleaning and shaping. Calcium hydroxide (Ca(OH)_2_) is the most common intracanal medication used, but it has several drawbacks such as inactivation by the buffering action of hydroxyapatite in dentin [6], reduction in compressive strength [7], difficulty in removal [8], and low efficiency on *E. faecalis* [9], which is commonly present in both primary (4–40%) and secondary/persistent apical periodontitis (44–77%) [10,11].

Complementary and alternative medicine (CAM) is an approach that uses systems, practices, and products that are not considered in conventional medicine. Phytotherapeutics are elements of CAM that rely on the use of herbal extracts to enhance the wellness of the individual [12]. The introduction of these herbs is related to their biocompatibility, antioxidant, anti-inflammatory, and antibacterial activities. *Salvadora persica* (miswak) belongs to the family Salvadoraceae [13]. *S. persica* (arak) is mainly present in the Arabian Peninsula, India, Iraq, Sri Lanka, Pakistan, and Africa [14]. *S. persica* was the first plant used in oral hygiene as a toothbrush, it is named the toothbrush tree (miswak) [15]. In 2000, WHO approved miswak as an effective and low-cost toothbrush, which can be used routinely [16]. A variety of studies found that the leaves of this plant can be used for their hypoglycemic, antiplasmodial, analgesic, diuretic, antiseptic, antifungal, antibacterial, and anticaries properties [17,18]. The stem of *S. persica* has profound antifungal, antimicrobial, and antiplaque actions [15,18]. *S. persica* consist of 1,8-cineole (eucalyptol) (46%), α-caryophellene (13.4%), β-pinene (6.3%), and 9-epi-(*E*)-caryophellene as major chemical structures [19]. Miswak contains multiple bioactive ingredients. The most important and effective ingredient is benzyl isothiocyanate, a major antimicrobial volatile oil [20]. Other valid ingredients that are present in *S. persica* include calcium, chloride, fluoride, vitamin C, *N*-benzyl-2-phenylacetamide, and flavonoids [14,21]. Calcium, chloride, and fluoride have anticariogenic properties and prevent the formation of calculus and tooth demineralization. The presence of vitamin C helps in healing and repair of the oral tissues; the vitamin also acts as an antioxidant [14,22]. This plant also contains *N*-benzyl-2-phenylacetamide, which has shown efficacy against *Escherichia coli* [23]. The endodontics literature elucidates the mutable uses of *S. persica* for root canal irrigation [24], smear layer removal [25], and intracanal medication [26]. No study in the literature has used *S. persica* extract at 100% concentration.

This study aimed to test the biocompatibility and antibacterial effect of 100% *S. persica* extract as an intracanal medication. Its effect is compared with that of Metapaste, which is commonly used for this purpose. The null hypothesis (HO) is that there would be no significant difference between *S. persica* and Metapaste in terms of pH, biocompatibility, antimicrobial properties, and extension of dentin disinfection activity.

## 2. Materials and Methods

**Figure 1:**
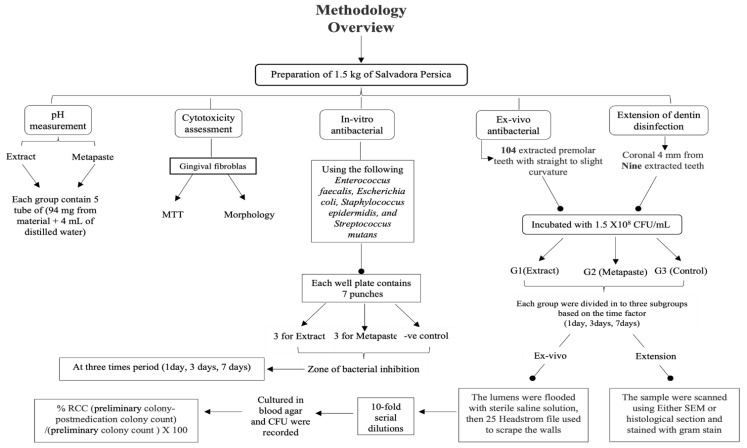
Summary of the methodology.

Figure 1 shows a summary of the study methodology. A research protocol was approved by the Research Ethics Committee at King Abdulaziz University, Faculty of Dentistry (approval no. 095-09-18). In this experiment, two materials were compared, a methanolic extract of *Salvadora persica* and Metapaste (META BIOMED, Cheongju, Korea). Metapaste is a conventional intracanal medication and it consists of calcium hydroxide as the basic ingredient with barium sulfate (radio-opacifier) and poly propylene glycol for material diffusion.

### 2.1. Preparation of the S. persica Methanolic Extract

The methanolic extract of *S. persica* plant was prepared at the Faculty of Pharmacy under the Pharmaceutical Consulting and Research Unit at King Abdulaziz University (KAU), Jeddah, Saudi Arabia. Fresh arak roots (*Salvadora persica*) (1.5 kg) were obtained from a traditional store, Jeddah city, Saudi Arabia, which were collected by an expert from the western region. The arak roots were minced and dried, pulverized, and extracted with methanol (3 × 1 L, Sigma-Aldrich, Chemie GmbH, Germany) using an ULTRA-TURRAX disperser (T 50 basic IKA-Werke, Germany) for 3–5 min to ensure an equal distribution of the methanol. The pooled methanolic extract was filtered until a clear solution was obtained and then evaporated under vacuum at 45 °C and 70–100 rpm using a rotary evaporator (Rotavap, BUCHI, Switzerland). The extracted product was then dried using a lyophilized freeze-dryer (Alpha 3-4 LSCbasic, CHRIST, Germany) at −80 °C for 3 days to obtain the crude extract. The extract was sterilized with UV light for 1 h.

### 2.2. pH Measurements

The pH was measured using a calibrated pH meter (Orion Star™ A214, Thermo Fisher Scientific) and checked with buffer solutions with pH values of 7 and 4. A total of 15 samples, in 5 mL polyethylene tubes, contained an equal amount of normal saline divided into three groups (Table 1). The pH of the solutions was measured at different time periods (0 h, 2 h, 4 h, 1 day, 3 days, and 7 days) [27] (Table 1).

### 2.3. Biocompatibility Assessment

After obtaining written consent from patients who had undergone endodontic microsurgery at the postgraduate endodontic clinic in the KAU Faculty of Dentistry, the granulation tissue attached to the root apex was taken for isolation of fibroblast cells. The primary culture of gingival fibroblasts was established as described by Kalamegam et al. in 2018 [28]. Cells at passages 5–8 were used for this experiment. This procedure was performed at the King Fahad Medical Research Center at KAU.

**Figure 2:**
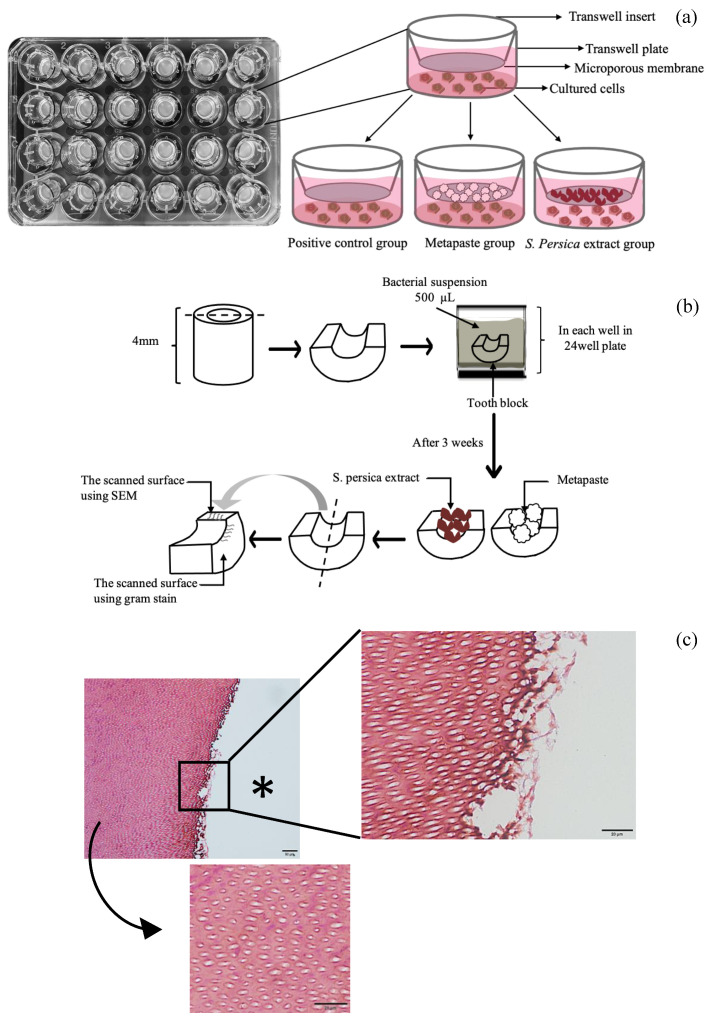
(**a**) The distribution of materials in the plate in the biocompatibility assessment. (**b**) Diagram illustrating the steps of sample preparation. (**c**) The two areas that were scanned for the presence of bacteria. The asterisk indicates the position of the canal lumen, the zoomed-in view is of the surface of the canal lumen, and the arrow indicates a view of the deeper region.

Cells were plated in a Transwell 24-well plate at a density of 5 × 10^4^ cells/well and incubated overnight in complete growth medium at 37 °C, 95% humidity, and 5% CO_2_. After 24 h, the wells divided in to three groups: group 1, *S. persica* (21 wells); group 2, Metapaste (21 wells); group 3, positive control (the cells left in culture medium only) (21 wells). Next, 100 mg of each material (*S. persica* extract and Metapaste) was placed on the Transwell insert. Then, 500 µL of complete Dulbecco’s modified Eagle medium (DMEM) was placed on each Transwell plate (Figure 2a). The attachment and viability were assessed using light microscopy and an MTT assay after 1, 3, and 7 days. The medium was changed every 2–3 days.

### 2.4. Cell Attachment

After each period, the Transwell insert was removed, the medium was aspirated, and the cultured cells were washed with 200 µL of phosphate-buffered saline. The cells were then visualized and photographed using a Nikon ECLIPSE TS 100 microscope (Nikon, Melville, NY, USA) at 4× and 10× magnifications. The morphological features and degree of confluence of the cells were examined and compared for each time period.

### 2.5. Cell Viability

Cell viability was tested using a (4,5-dimethylthiazol-2-yl)-2,5-diphenyltetrazolium bromide (MTT) assay. At each time period, the tissue medium was removed, followed by the addition of 50 µL of MTT working solution (final concentration 5 mg/mL) and 450 µL of complete medium to each well after removing the Transwell insert and washing of the cells with 200 µL of phosphate-buffered saline. The reagent was incubated for 3–4 h at 37 °C, 95% humidity, and 5% CO_2_. After the incubation period, 50 µL of dimethyl sulfoxide (DMSO) solution was added. After 10 min, 100 µm from each well was transferred into a 96-well plate, and the absorbance was read at 570 nm using a Microplate reader (SpectraMax i3, Molecular Devices, San Jose, CA, USA). To assess cell viability, the following formula was used [29,30]:

$$\frac{Mean\;of\;the\;sample}{Mean\;of\;the\;control\;}\times 100\;$$

Results were interpreted as follows: no cytotoxicity if viability was >90%; slight cytotoxicity if viability was 60–90%; moderate cytotoxicity if viability was 30–59%; strong cytotoxicity if viability was <30%.

### 2.6. In Vitro Antibacterial Activity (Agar-Well Diffusion Test)

Four common types of intracanal bacteria were used in this study: *Enterococcus faecalis* (*E*. *faecalis*) (ATCC 29212), *Escherichia coli* (*E. coli*) (ATCC 25922), *Staphylococcus epidermidis* (*S. epidermidis*) (ATCC 12228), and *Streptococcus mutans* (*S. mutans*) (ATCC 25175). Plates were contaminated with different bacteria at a concentration of 1.5 × 10^8^ colony-forming units (CFU)/mL and adjusted to 0.5 McFarland; one plate was used for each bacterial type. Seven punches with a depth of 5 mm and diameter of 4 mm were prepared using a premeasured sterile stainless-steel tube. Three wells were filled with Metapaste, and the other three were filled with *S. persica* extract. The middle well was left empty, as a negative control. The zone of bacterial inhibition was measured by recording the largest diameter of inhibition surrounding each punch in mm using a ruler [31] at three different time periods (1, 3, and 7 days).

### 2.7. Ex Vivo Antibacterial Activity (% Reduction in Colony Count)

A total of 104 extracted intact single-rooted human permanent premolar teeth with a straight to slightly curved root were selected and stored in normal saline to prevent dehydration. Teeth were decoronated and sectioned to a working length of 15 mm, and then the Reciproc rotary files were used up to R40 to instrument the canals 0.5–1 mm beyond the apex. Subsequently, the enlarged apexes were sealed with an epoxy resin. Samples were agitated using an ultrasonic path with 5.25% NaOCl for 5 min, followed by 17% EDTA for 5 min, and then distilled water was used for 10 min [32]. The roots were coated from the external surface using nail polish to avoid external bacterial contamination [33]. Then, they were mounted vertically inside a 5 mL polyethylene tube using a heavy putty impression material. These samples were sterilized at 121 °C for 36 min. Two samples were cultured for a sterility check. A pure culture of *E*. *faecalis* (ATCC 29212) was selected. The colonies were suspended in TSB to obtain 1.5 × 10^8^ CFU/mL and adjusted to 0.5 McFarland [33]. Then, each root was contaminated with 20 μL of suspension containing the tested organism. Next, the roots were incubated at 37 °C for 3 weeks. In a laminar air-flow cabinet, the broth in each root canal was refreshed every 48 h. To verify biofilm formation, two roots were split vertically into two segments and scanned with a scanning electron microscope (SEM) (AURA 100, Saeron Technology, Korea). After 3 weeks of incubation, the samples were randomly divided into three groups: group 1, *S. persica* extract (*n* = 39); group 2, Metapaste (*n* = 39); group 3, positive control (canals left empty) (*n* = 24). For the *S. persica* extract, the material was placed on top of the canal with a spoon excavator and then condensed using a spreader to ensure that it reached the full length of the canal. All three groups were subdivided into three subgroups on the basis of the three time periods: 1, 3, and 7 days. All roots were incubated at 37 °C.

For each root in each subgroup, two bacterial samples (preliminary S1 and post-medication sample S2) were collected. S1 was taken after the incubation period and before the application of either *S. persica* extract or Metapaste [32]. For sample preparation (S1, S2), the lumens were flooded with sterile saline solution, and then 25 Headstrom files were inserted and moved in an up-and-down motion to gently scrape the canal walls. Then, a sterile paper point was placed inside the canal and left for 60 s to absorb the canal contents. The paper point was then transferred to a test tube containing 1.0 mL of sterile normal saline and shaken using a vortex mixer for another 60 s. Tenfold serial dilutions were prepared from the original shaken suspension. A 10 μm disposable inoculating loop was then inserted inside each dilution and transferred to sheep blood agar for manual plating and incubated aerobically at 37 °C for 24 h. After the incubation period, CFUs were recorded using a colony-counting device.

The percentage reduction in colony count (% RCC) was calculated using the following formula [32]:

(1) $$\%\;RCC=\frac{Preliminary\;colony-postmedication\;colony\;count\;}{Preliminary\;\;colony\;count\;}\times 100$$

### 2.8. Extension of Dentin Disinfection Activity

To check the extent of antibacterial activity of the materials, SEM and light microscopy were used. Root dentin blocks [34] were prepared from nine extracted premolar single-rooted teeth. Four mm thick coronal dentin blocks were cut. The blocks were then enlarged to 1.5 mm diameter using Gate Glidden drill #6. Each block was then sectioned into two semicylindrical halves using a diamond disc. The outer layer of each section was ground to achieve a thickness of 2 mm and to remove the outer cementum layer. Then, the samples were soaked in an ultrasonic path following the same protocol as in the ex vivo experiment. The outer surface of the blocks was covered with nail varnish. The samples were then autoclaved at 121 °C for 36 min.

A pure culture of *E. faecalis* (ATCC 29212) was selected. The same bacterial suspension prepared in the ex vivo experiment was used. Then, 500 µL of the prepared suspension was added to 24-well plates containing the dentin block and incubated for 3 weeks. The medium was changed every 48 h.

After the incubation period, the blocks were rinsed with sterile water for 1 min and then divided into three main groups: group 1, *S*. *persica* extract (*n* = 6); group 2, Metapaste (*n* = 6); group 3, positive control (root segment left without any medication) (*n* = 3). Each group was subdivided into three subgroups on the basis of the three experimental time periods.

After each period, the samples were washed with sterile normal saline and stored in 10% neutral buffered formalin for a minimum of 24 h. Then, each block (semicylindrical half) was cut into two halves (quarter), and the inner surface was inspected using either SEM to confirm the presence and penetration of bacteria or stained with Gram stain for histological examination (Figure 2b).

### 2.9. Scanning Electron Microscopy

One-quarter of each sample was taken from each group, including the positive control, and scanned using SEM (AURA 100, Saeron Technology, Korea). The samples were soaked in 100% ethanol for 10 min. The segments were sputter-coated with gold/platinum for 180 s in a coating machine (Quorum SC7620, Quorum Technologies, UK).

### 2.10. Gram Staining Technique

Another quarter was assigned for Gram staining. The staining process was carried out as follows: demineralization in 40% hydrochloric acid (TBD-1) for 4–5 h. Then, the samples were hydrated using an ascending grade of ethanol. This was followed by clearing using xylene to allow the infiltration of the paraffin wax (melting point 56 °C). Samples were positioned in such a way that the cross-section of the root segment was parallel to the floor and then embedded in paraffin wax. Using a microtome, 3 µm histological sections were prepared. Nine sections were obtained from each sample. For staining, a Gram stain kit was used (CRESCENT Diagnostics, Jeddah, Saudi Arabia), and the manufacturer’s instructions for slide preparation were followed. Slides were examined using a Nikon ECLIPSE TS 100 microscope (Nikon, Melville, NY, USA) at a magnification of 10× and 40× to trace the area of blue/black dots that filled and attached to the lumen of the dentinal tubules, which represented the presence of Gram-positive bacteria (*E. faecalis*) (Figure 2c).

### 2.11. Statistical Analysis

Data were analyzed using SPSS version 25 (IBM Corp., Armonk, NY, USA). Normality was checked. For comparison of means, the Wilcoxon signed-rank test, Mann–Whitney U-test, and Kruskal–Wallis test were used.

## 3. Results

### 3.1. pH Measurements

Table 2 shows the results of the *S. persica* extract and Metapaste. The extract showed an acidic pH, whereas Metapaste showed an alkaline pH. There was a highly significant difference in pH among the three groups (*p* ≤ 0.05).

### 3.2. Biocompatibility Assessment

#### 3.2.1. Cell Morphology

**Figure 3:**
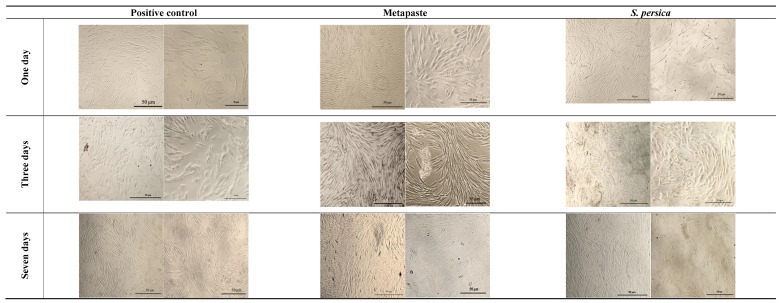
Light microscopy images of human gingival fibroblasts in the control, Metapaste, and *S. persica* groups after 1, 3, and 7 days of exposure, at 4× magnification power and 10× magnification power. The scale bar of all images is 50 μm.

Figure 3 shows the morphology of human gingival fibroblasts grown in the presence of both *S. persica* and Metapaste at different timepoints in comparison to the positive control cells. As observed, cells grown in the presence of *S. persica* and Metapaste showed a spindle-shaped morphology similar to that of the positive control cells. For all groups, the number of cells increased with time, as indicated by increased cell density, and the cells were connected by their cytoplasmic processes.

#### 3.2.2. Cell Viability (MTT Assay)

**Figure 4:**
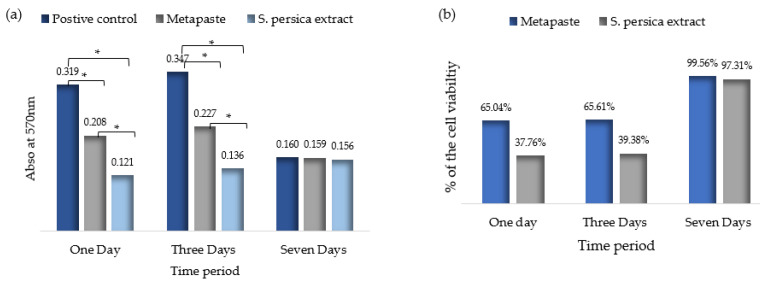
(**a**) MTT assay for human fibroblasts; (**b**) viability of fibroblast cells after different time periods. * Denote the statistical significant within the same time periods.

Cell viability was estimated on the basis of the absorbance at 570 nm at *p* ≤ 0.05 (Figure 4). There was a statistically significant difference between all groups at 1 and 3 days only. An increase in cell viability in the *S. persica* group was recorded at 3 and 7 days, with only a statistically significant difference between days 1 and 3 (*p* ≤ 0.05). In contrast, in the Metapaste group, cell viability increased at 3 days and then dropped at 7 days, with no statistically significant difference at any timepoint (*p* > 0.05). For the control group, a significant change in cell viability was observed after 7 days (*p* ≤ 0.05). Overall, among the three experimental groups, no significant differences were found after 7 days (Figure 4a). Metapaste showed slight cytotoxicity at 1 and 3 days, whereas *S. persica* showed moderate cytotoxicity. At 7 days, neither material was cytotoxic (Figure 4b).

### 3.3. In Vitro Antibacterial Activity

**Figure 5:**
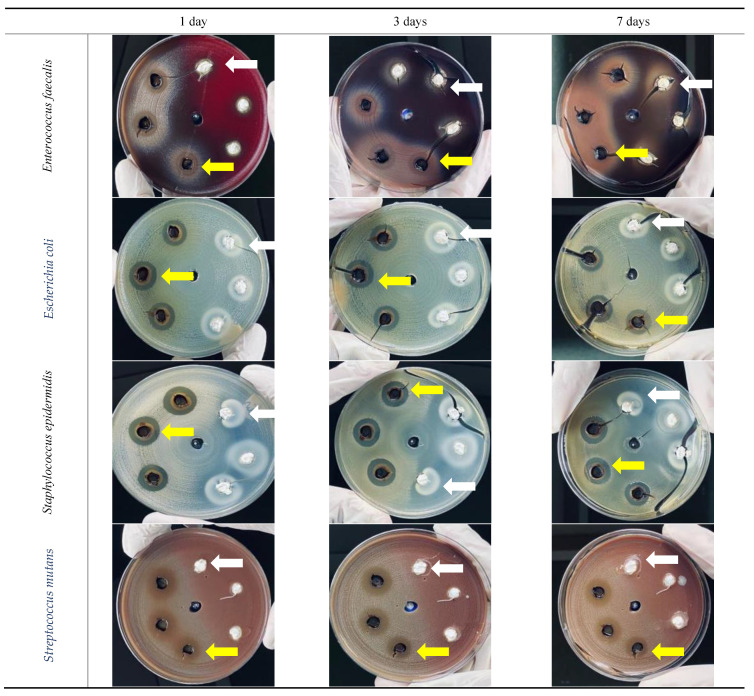
Inhibition zone of the growth of four bacteria by *S. persica* extract (yellow arrows) and Metapaste (white arrows) after 1, 3, and 7 days.

The results of the agar well diffusion test are shown in Table 3 and Figure 5.

#### 3.3.1. *Enterococcus faecalis*

With the *S. persica* extract, the largest zone of inhibition was observed after 3 days (15 mm diameter). After 7 days, it returned to the same level as that on 1 day (13.3 mm). Metapaste did not show any effect against *E. faecalis* even after 7 days. The mean zone of inhibition at all three timepoints for Metapaste was statistically significantly different from that of *S. persica* (*p* ≤ 0.05).

#### 3.3.2. *Escherichia coli*

The effect of *S. persica* extract on *E. coli* reached the maximum level (15.3 mm) after 3 days and was maintained after 7 days. In contrast, this organism showed resistance toward Metapaste on the first day; then, after 3 days, it started to show a zone of bacterial inhibition with a mean size of 16 mm. After 7 days, the inhibition zone decreased to 13.7 mm with only a statistically significant difference between both materials after 1 day (*p* ≤ 0.05).

#### 3.3.3. *Staphylococcus epidermidis*

The effect of *S. persica* extract reached its maximum after 3 days with an average 17.3 mm of inhibition zone. This was also noted when using Metapaste with a mean of 17.7 mm. With both *S. persica* and Metapaste, the inhibition zone was reduced after 7 days to 16.3 mm and 16.7 mm, respectively, with no statistically significant difference between the two materials (*p* > 0.05).

#### 3.3.4. *Streptococcus mutans*

With *S. persica* extract, the largest mean inhibition zone (15 mm) appeared on day one. This effect was maintained after 3 days and then reduced after 7 days (14.7 mm). On the other hand, the application of Metapaste to this strain showed no effect even after 7 days. The difference between the two materials was statistically significant at all timepoints (*p* ≤ 0.05).

### 3.4. Ex Vivo Antibacterial Activity

**Figure 6:**
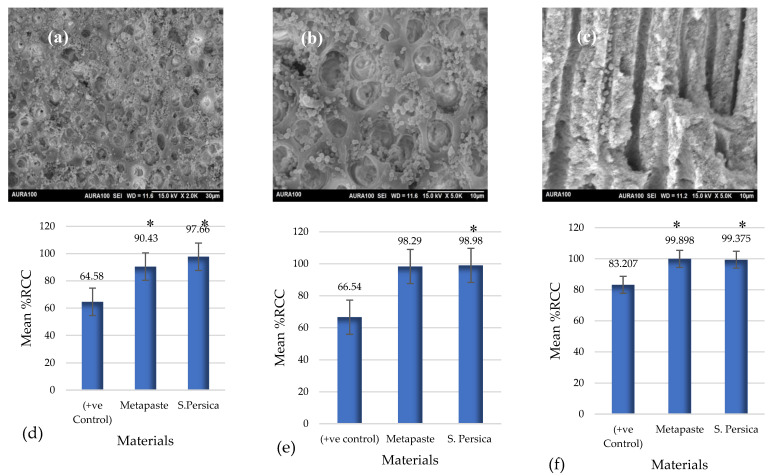
(**a**–**c**) SEM images of biofilm formation: (**a**,**c**) show the inner wall of the canal lumen, while (**b**) shows the side of the canal lumen. (**d**–**f**) Mean % RCC of *Enterococcus faecalis* in *S. persica* and Metapaste group in comparison to the positive control after 1 day (**d**), 3 days (**e**), and 7 days (**f**). * Denote the statistical significant within the same time periods.

SEM analysis showed a dense bacterial infection in the outer wall of the canal lumen (Figure 6a–c).

A significant change in the CFU/mL between the pre-treatment and post-treatment groups in all tested materials at the three timepoints (*p* ≤ 0.05) was observed. The only exception was the positive control group at 1 and 7 days (*p* > 0.05).

Regarding the percentage reduction in colony count (% RCC) at various time intervals (Figure 6d–f), after 1 day, the maximum reduction in *E. faecalis* colonies was observed in *S. persica* (97.66%) while the minimum amount was observed in the control group (64.5%). Using the Mann–Whitney U-test, a statistically significant difference was found between the positive control and both Metapaste and *S. persica* extracts (*p* ≤ 0.05). No statistically significant differences were found between the Metapaste and *S. persica* extracts. At 3 days, the maximum reduction in *E. faecalis* colonies was found in *S. persica* (98.9%), while the minimum was observed in the control groups (66.5%). Mann–Whitney U-test analysis showed no statistically significant difference in the % RCC between the positive control and Metapaste groups (*p* > 0.05), while a statistically significant difference was observed between the control and *S. persica* extract groups (*p* ≤ 0.05). No significant difference was observed between the Metapaste and *S. persica* extract groups. After 7 days, the maximum reduction in the *E. faecalis* colony was found in the Metapaste group (99.8%), while the lowest was observed in the positive control group (83.2%). According to the Mann–Whitney U-test, the mean % RCC in the control group was statistically significant compared to that in the Metapaste and *S. persica* extracts (*p* ≤ 0.05). There was no significant difference between the Metapaste and *S. persica* extracts (*p* > 0.05). Generally, the maximum % RCCs for both Metapaste and *S. persica* were observed after 7 days.

### 3.5. Extension of Dentin Disinfection Activity

#### 3.5.1. Scanning Electron Microscopy

**Figure 7:**
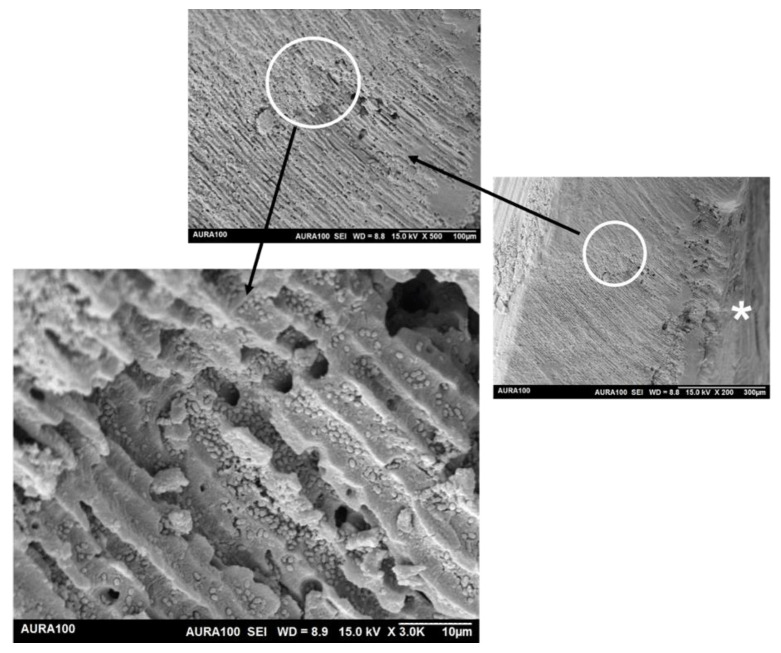
The maximum penetration depth of bacteria inside the dentinal tubules. The asterisk indicates the position of the canal lumen.

SEM showed that the maximum penetration depth of *E. faecalis* inside the dentinal tubules reached >300 µm (Figure 7).

#### 3.5.2. Gram Staining

Using the Gram staining technique, the presence of bacteria in the positive control group, indicated by the purple dots, and its penetration were obvious at all timepoints but tended to decrease with time, especially on the surface of the canal lumen. In the Metapaste group, after 1 and 3 days, no bacteria were found on the surface of the canal lumen. However, in the deeper part of the specimen, the presence of bacteria was obvious. After 7 days, no bacteria were found in the deeper part, and only a few were scattered on the surface of the canal lumen.

**Figure 8:**
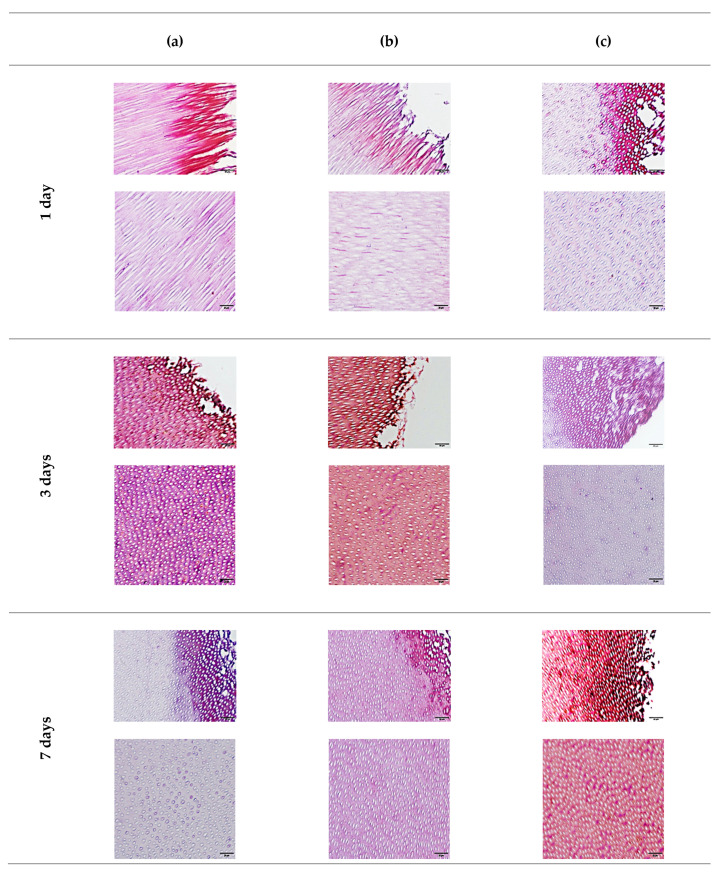
Light microscopic images of Gram-stained sections for positive control (**a**), Metapaste (**b**), and *S. persica* (**c**), after 1 day, 3 days, and 7 days, at 40× magnification. The scale bar of all images is 20 μm.

In the *S. persica* extract, after 1 day, few bacteria were present on the surface of the canal lumen, but more bacteria were observed in the deeper part. After 3 days, the bacteria disappeared on the surface of the canal lumen and their number was reduced in the deeper part. Finally, after 7 days, no bacteria were found either on the surface of the canal lumen or in the deeper part (Figure 8).

## 4. Discussion

The main goal of root canal treatment is to reduce intraradicular microorganisms to a subclinical level [35]. Multiple attempts have been made to overcome the drawbacks associated with some conventional intracanal medications. One of these trials was to utilize nanotechnology in conventional antibacterial medications. Silver nanoparticles (AgNPs) are well known to have high efficiency against Gram-positive and Gram-negative bacteria [36]. The incorporation of this material with a conventional one (Ca(OH)_2_) as intracanal medication showed better antibacterial activity against the resistant strain [33]. Zinc oxide nanoparticles added to the Ca(OH)_2_ also increased the antibacterial effect and the penetrability [37,38]. A debate exists around the safety of nanotechnology and the effect of these products on general health [39]. This is supplemented by the complexity of production methods. Another attempt was to utilize the concept of CAM in the field of endodontics, which is a less sensitive and less expensive technique. Miswak is a well-known plant, especially in the Middle East, which is used for oral hygiene due to its antibacterial activity [40].

Two main techniques have been reported for the preparation of *S. persica* extract, using either alcohol or water extraction methods. In this study, an alcoholic extraction method was employed. Abdallah and Al-Harbi (2015) investigated the phytochemical constituents of *S. persica* and found that the ethanolic extraction method is characterized by flavonoids, which are known to interact with the bacterial cell wall and inhibit bacterial growth as end-products [41]. Al-Ayed et al. (2016) compared methanolic extracts with water extracts by measuring the zone of growth inhibition and found that the methanolic extract has a more potent antibacterial effect against resistant strains such as *E. faecalis* than the aqueous extract [42]. On the other hand, Al-bayati et al. (2008) found that the aqueous extract is more antibacterial than the alcoholic extract [43]. The diversity in the results for the antibacterial effect of both methods may be related to the geographic origin of the plants [27]. Furthermore, the alcoholic method is safer and more popular due to its low cost [44]. In this article, the extract was sterilized using UV light at a wavelength of 100–280 nm. This technique can be used to sterilize the extract without changing its physical structure. Some studies have shown that UV radiation in the range of 280–300 nm leads to changes in the plant [45,46] In another study, the small UV wavelength was found to reduce the flavonoid composition in the plant extract after short-term exposure [47]; no studies assessed the effect of UV light on *S. persica* extract.

The physical properties, precisely the pH, of intracanal medication play a role in the antibacterial action of some medicaments. Furthermore, the pH of the root canal medications might have an effect on dissolution of the mineralized components of the teeth. In our experiment, *S. persica* was more acidic in nature, whereas Metapaste was alkaline. The acidity of *S. persica* could be attributed to its content of oleic, linoleic, and stearic acids [25]. Adding to this is the acidic nature of alcoholic extraction methods [48].

For cytotoxicity assessment, cells derived from periapical granulation tissues, which are the cells with which the degradation products of any intracanal medication will react, were used. In addition, the cytotoxicity test was performed by applying the material in a Transwell plate to test the indirect effect of the intracanal medications (Metapaste and methanolic extract of *S. persica*) to simulate the clinical application.

The MTT assay based on ISO 10993-1 was used [49]. According to the results of this study, on the first day, both the *S. persica* and the Metapaste samples showed very low cell viability (37% and 65%, respectively). The cell viability then increased with time. For *S. persica*, our findings are in agreement with the study conducted by Tabatabaei et al., who found that, within 24–48 h of applying an increasing concentration of ethanolic extract, the cell viability was reduced to 70% compared to the cells with medium only [50]. The opposite was found by Balto et al., where the ethanolic extracts of *S. persica* at concentrations of 0.5 and 1 mg/mL were not cytotoxic after only 1 day of exposure [51]. Balto et al. used diluted ethanolic extracts; however, in this study, a 100% extract was prepared by applying the dry extract without adding any solvent.

Reduced cytotoxicity of *S. persica* over time was also observed in our experiment. It has been documented that the toxicity of *S. persica* occurs due to irreversible binding with cell proteins [50]. The reduction in the cytotoxicity of *S. persica* over time could be attributed to the binding of the toxic material in *S. persica* with the serum in the tissue culture medium [52]. In addition, we observed an increase in cell viability at 3 and 7 days after application of *S. persica*, which is similar to what was found by Darmani and Al-Hiyasat [53]. Regarding Metapaste, the highest toxicity was observed after 1 day, which was reduced with time, and cell viability increased. These findings are in agreement with those of Labban et al. [30]. The main cause for the reduction in toxicity of Metapaste is the dilution of the material by the action of the cultured medium. This dilution effect could be responsible for the reduction in the alkalinity of Metapaste, which could explain the improvement in cell viability and the decrease in toxicity seen over time. Regarding the control cells, after 7 days, the reduced cell viability could be explained by a density-dependent inhibition of cell growth [54].

To test the biofilm removal ability of any root canal-related materials, several techniques were used: culture technique, histobacteriological approach, and SEM. The culturing technique aimed to remove, count, and culture most of the bacteria and bacterial biofilms present in the main canal. The culturing technique has been used traditionally for testing intracanal bacteria for more than a century. Although it is considered the most famous technique for bacterial detection, it has several drawbacks, such as technique sensitivity, especially for anaerobic bacteria [55].

In our in vitro experiment, four types of microorganisms commonly present in oral infections were used [56]. *E. coli* is not commonly present in endodontic infections, but its lipopolysaccharides are used as a reference to test the antibacterial effect of medication, since it is present in the majority of Gram-negative bacteria [57]. The results showed that both materials had an inhibitory effect against *E. coli* and *S. epidermidis*, whereas *S. mutans* and *E. faecalis* only showed an inhibition zone with *S. persica* extract. Testing the antimicrobial effect of any material with an agar diffusion test depends on the diffusion of this material in the agar plate [58]. Therefore, the weak effect of Metapaste against *E. faecalis* in this test was due to weak diffusion ability in the agar material and the neutralization of the Metapaste by the agar, which lowered its pH [59].

Regarding the effect of *S. persica*, our findings are in agreement with Balto et al., who found that the ethanolic extract showed an increased inhibitory effect against *S. mutans* after 48 h of application [60]. For its effect with *E. faecalis*, a study conducted by Gupta et al. in 2020 showed that 12.5% ethanolic extract of *S. persica* had an acceptable antibacterial effect when tested by agar well diffusion methods against *E. faecalis*, but 5% sodium hypochlorite showed a higher zone of inhibition with statistical significance [61].

To test our experimental materials as intracanal medication, a single-species biofilm of *E. faecalis* was selected for the ex vivo study. Our study focused on one of the Gram-positive bacteria because Gram-negative bacteria are easily eliminated during instrumentation and irrigation, while Gram-positive bacteria are more resistant [62]. *E. faecalis* was selected because it can form a biofilm, penetrates deeply into the dentinal tubules [63], can withstand a long starvation period [64], is resistant to high alkalinity, and is highly expressed in persistent infection [65]. The incubation period of the bacteria was 3 weeks, as this is the time required for *E. faecalis* to reach its maximum penetration depth [66]. Furthermore, 3 week old bacterial biofilms are more resistant to antimicrobial agents than 1 or 2 week old biofilms [67]. According to the results of this study, both *S. persica* and Metapaste showed a significant difference in the amount of bacteria reduction compared to the control at the three timepoints.

Our results are relatively similar to those of a study by Madhubala et al., who found that Ca(OH)_2_ is an effective antimicrobial medication from the first day and the effectivity increased after 2 and 7 days, but the amount of bacteria reduction was very low (25%, 39%, and 59%, respectively) when compared to our experiment [32]. To our knowledge, only one study tested the *S. persica* extract as a root canal medication, and they found that an 8% concentration of *S. persica* significantly reduced the bacterial count [26]. In this previous study, diluted concentrations from *S. persica* (2%, 4%, and 8%) were used, and the results were compared with those of chlorhexidine intracanal paste, which is not a regular material used in our daily practice. The researchers measured the CFU/mL after 7 days only.

The penetration of *E. faecalis* inside dentinal tubules is an important mechanism for bacterial resistance [68]. Therefore, testing the penetration depth of any introduced antimicrobial agent is crucial. The dentin block model can provide predictable, dense, and deep penetration of the bacteria, which may reach up to 500 µm [34,66]. To the best of our knowledge, no study has tested the extension of the antimicrobial effects of *S. persica* extract inside the dentinal tubules. Our results showed that the diffusion of Metapaste in dentin was faster than that of *S. persica*, which was confirmed by the absence of bacteria on the surface of the canal lumen in the Metapaste group after 1 day only. The acceptable diffusion ability of Metapaste is due to the presence of propylene glycol as a vehicle; this material proved to provide the Metapaste with good diffusion in the dentin [69]. The results also showed that after 3 and 7 days, the effect of penetration of *S. persica* was comparable to that of Metapaste. The limitations of this experiment include (a) the type of bacterial strains that we used, the laboratory reference strain, which had weak pathogenicity compared to the clinically isolated strains [70], (b) the presence of a monospecies biofilm which is rarely applied in real life [71], and (c) the acidic nature of the extract, which may have had a side-effect on the tooth structure. Future research attempts should be made to try reduce the acidity of this extract to make it safer for dentin and to test its effect on the dentin structure.

## 5. Conclusions

The results of these in vivo and ex vivo experiments indicated that the 100% concentration of *S. persica* methanolic extract has effective antimicrobial activity. Its antimicrobial activity is based on its composition due to the presence of toxic products that bind to the cell wall. Furthermore, the antimicrobial property is not dependent on the pH of the material, as is the case with Metapaste, which can be neutralized by the action of some bacterial species. In addition, *S. persica* extract showed lower cell viability in the initial period.

## Figures and Tables

**Table 1:** Study groups for the pH measurement.

Study Group	Group Discription
Group 1	94 mg of *S. persica* extract in 4 mL of normal saline (*n* = 5)
Group 2	94 mg of Metapaste in 4 mL of normal saline (*n* = 5)
Group 3	4 mL of normal saline (*n* = 5)

**Table 2:** Mean ± SD for the pH of the three groups at different timepoints.

Time Points	0 h	2 h	4 h	1 Day	3 Days	7 Days
*S.persica* extract	3.79 ± 0.36 c	3.79 ± 0.38 c	3.83 ± 0.40 c	3.91 ± 0.37 c	3.93 ± 0.37 c	3.75 ± 0.39 c
Metapaste	12.42 ± 0.11 a	12.42 ± 0.09 a	12.39 ± 0.08 a	12.51 ± 0.03 a	12.48 ± 0.13 a	12.40 ± 0.21 a
Positive control	5.40 ± 0.07 b *	6.04 ± 0.41 b	6.24 ± 0.50 b	5.89 ± 0.32 b	6.04 ± 0.30 b	6.13 ± 0.40 b

The significance was obtained by using a Kruskal–Wallis test and post hoc tests (Mann–Whitney U) at *p* ≤ 0.05. Different letters denote a statistically significant difference between the materials. * Statistically significant difference within the same material.

**Table 3:** Mean ± SD of the inhibition zone of four tested bacterial types.

Bacterial Type	*E. faecalis*			*E. coli*			*S. epidermids*			*S. mutans*		
Time period (Days)	1	3	7	1	3	7	1	3	7	1	3	7
*S. Persica* extract (± SD)	13.3 ± 1.2 a	15 ± 1.7 a	13.3 ± 1.2 a	14.3 ± 0.6 a	15.3 ± 1.5	15.3 ± 1.2	16.6 ± 0.6	17.3 ± 1.2	16.3 ± 1.2	15 ± 2.6 a	15 ± 1 a	14.6 ± 1.5 a
Metapaste (± SD)	0	0	0	0	16 ± 2	13.6 ± 0.6	18 ± 2	17.6 ± 0.6	16.6 ± 2.3	0	0	0

Significance was obtained using the Kruskal–Wallis test and post hoc tests (Mann–Whitney U) at *p* ≤ 0.05. Different letters denote a statistically significant difference between the experimental materials within the same period.

## Data Availability

Data sharing not applicable. No new data were created or analyzed in this study. Data sharing is not applicable to this article.
